# Two new species of *Desmopachria* Babington, 1841 in the *D. convexa* species group (Coleoptera, Adephaga, Dytiscidae, Hydroporinae, Hyphydrini)

**DOI:** 10.3897/zookeys.923.47104

**Published:** 2020-04-01

**Authors:** Kelly B. Miller

**Affiliations:** 1 Department of Biology and Museum of Southwestern Biology, University of New Mexico, Albuquerque, NM 87131-0001 USA University of New Mexico Albuquerque United States of America

**Keywords:** Taxonomy, New World, diving beetles, systematics

## Abstract

Two new species are described in the *Desmopachria convexa* species group in the Neotropical genus *Desmopachria* Babington: *D. manco***sp. nov.** (Guyana), and *D. mortimer***sp. nov.** (Costa Rica). Two subgroups, the *D. convexa-convexa* and the *D. convexa-signata* groups are defined. *Desmopachria convexa-convexa* species are from North and Central America and have a subapical articulable lobe on the male lateral lobe that is large and elongate and extends well beyond the slender, oblique apex of the lateral lobe. *Desmopachria convexa-signata* species are from South America and have a subapical articulable lobe on the male lateral lobe that is small and discrete and does not extend beyond the truncate apex of the lateral lobe. The male genitalia of all recognized species in the *D. convexa* group are redrawn from the literature. New species are illustrated from specimens and described species have morphological features redrawn from published illustrations.

## Abstract (es)

Se describen dos especies nuevas en el grupo de especies *Desmopachria convexa* del género neoprópical *Desmopachria* Babington: *D. manco***sp. nov.** (Guyana) y *D. mortimer***sp. nov.** (Costa Rica). Se definen dos subgrupos, el *D. convexa-convexa* y *D. convexa-signata*. Las especies de *D. conveza-convexa* son de Centro y Norte América, y tienen un lóbulo subapical articulado en el lóbulo lateral del macho que es grande y alargado y se extiende mucho más allá del ápice delgado y oblicuo del lóbulo lateral. Las especies de *D. convexa-signata* son de América del Sur y tienen un lóbulo subapical articulado en el lóbulo lateral del macho que es pequeño y discreto y no se extiende más allá del ápice del lóbulo lateral. Los genitales masculinos de todas las especies reconocidas en el grupo *D. convexa* se vuelven a dibujar a partir de la literatura. Las especies nuevas se ilustran a partir de los ejemplares examinados y las especies descritas tienen características morfológicas redibujadas a partir de ilustraciones publicadas.

## Introduction

The taxonomic situation concerning *Desmopachria* Babington was briefly reviewed most recently by Miller and Wolfe (2018; 2019) and Braga & Ferreira Jr. (2018). *Desmopachria* is a species-rich genus of diving beetles restricted to the New World with numerous species described recently (Braga and Ferreira-Jr. 2010; 2011; 2014; Gustafson and Miller 2012; Makhan 2012; 2015; Megna and Sanchez-Fernandez 2014; Miller 1999; 2001; 2005; Miller and Wolfe 2018; 2019). Currently the genus includes approximately 130 described species, and many more undescribed ones known to exist making it one of the larger genera of diving beetles in the New World.

Two new species are described here from the *D. convexa* species group which are characterized by an articulable appendage on the anterolateral surface of the male lateral lobe (Young 1980; 1981). This group was reviewed by Young (1981) with a number of species described subsequently (Braga and Ferreira-Jr. 2010; Miller 2001; 2005; Young 1990). The group is among the most widespread in *Desmopachria*, occurring throughout eastern North America south into southern South America.

Dichotomous keys are not particularly useful within *Desmopachria*, including the *D. convexa* group. The best strategy for identification of these extremely similar species is comparison of visual diagnostic combinations especially comparison of male genitalia with others in the group (Figs 2–5, 7–8, 11–36, 39–41, 44–46, 49–57).

## Material and methods

### Measurements

Measurements were made with an ocular scale on a Zeiss Discovery V8 dissecting microscope to 0.1 mm. The diagnostic range of measurements of structures was emphasized, so the largest and smallest specimens were preferentially measured to the extent possible. Measurements include: 1) total length (TL), 2) greatest width across elytra (GW), 3) greatest width of head (HW), and 4) distance between eyes (EW). The ratios TL/GW and HW/EW were also calculated.

### Images

Illustrations were made using a drawing tube on a Zeiss Discovery V8 dissecting scope. Sketches were first done in pencil then scanned, placed into an Adobe Illustrator artboard and “inked” digitally using vector lines and modified with brushes.

### Material

Specimens of *Desmopachria* were examined representing many species from all species groups including many from the following collections:

**CSBD**Center for Biological Diversity, University of Guyana (type specimens currently reposed with KUNHM, see below)

**KBMC**Kelly B. Miller Collection, Museum of Southwestern Biology, University of New Mexico, Albuquerque, NM, USA.

**KUNHM** University of Kansas Natural History Museum, University of Kansas, Lawrence, Kansas, USA (A.E.Z. Short, curator)

**MIZA**Museo del Instituto de Zoología Agrícola Francisco Fernández Yépez, Universidad Central de Venezuela, Maracay, Venezuela (L. Joly, curator)

**MSBA**Museum of Southwestern Biology Division of Arthropods, University of New Mexico, Albuquerque, NM, USA (K.B. Miller, curator)

**NZCS** National Zoological Collection of Suriname, Paramaribo, Suriname (P. Ouboter, curator)

**USNM**United States National Collection of Insects, Smithsonian Institution, Washington, DC, USA (T. Erwin, curator)

## Taxonomy

### The Desmopachria convexa

Taxon classificationAnimaliaColeopteraDytiscidae

group

72B3E65F-87F9-556A-BBEF-581CB3A0DDEF

#### Diagnosis.

The *Desmopachria convexa* group is characterized in the genus by an articulable subapical process on the male lateral lobe of the aedeagus and the male median lobe either apically bifid (e.g., Fig. 15) or trifid (e.g., Fig. 11) with the exception of *D. pilosa* Miller (apically simple, Fig. 52) and *D. majuscula* Young (seemingly absent, Fig. 34). The species are extremely similar to each other in external appearance, though there are some diagnostic variations in size, shape, punctation and coloration. But externally there are often few particularly useful characters for distinguishing closely related species. Males and females are externally extremely similar, as well.

There are two apparent subgroups in the *D. convexa* species group, those with a smaller subapical articulable appendage on the lateral lobe not extending beyond the truncate apex (e.g., Figs 4, 5) and those with a larger subapical articulable appendage that is leaf-like and extends well beyond the elongate, slender oblique apex of the lateral lobe (e.g., Figs 9, 10). These are referred to here as the *D. convexa-convexa* subgroup (with the larger subapical articulable appendage) and *D. convexa-signata* subgroup (with the smaller subapical articulable lobe). *Desmopachria convexa-convexa* species are found in North and Central America and the Caribbean, and *D. convexa-signata* species are found in South America. It is not clear at this time how these two groups might be related to each other or their monophyletic status, but they seem to be well-characterized by the shared articulable appendage of the male lateral lobes which is unique in *Desmopachria* and Dytiscidae in general.

#### Comments.

This group corresponds to the *Desmopachria convexa-grana* group of Young (1980), which he later revised (Young 1981). Additional new species were described by several investigators (Braga and Ferreira-Jr. 2010; Miller 2001; 2005; Young 1990).

It is possible that several other described species may belong to this species group including *D. attenuata* Régimbart, 1895 (Young 1980), *D. balfourbrownei* Young, 1990, *D. striga* Young, 1990, and *D. subfasciata* Young, 1990 based on illustrations suggesting the presence of a subapical or apical articulable structure on the lateral lobe (Miller 2001). These species have not been well-described making the diagnostic characteristics of the group hard to discern. It does not appear that these species correspond with either of the new species described here, however.

### Desmopachria manco sp. nov.

Taxon classificationAnimaliaColeopteraDytiscidae

9CD65445-421C-5F8C-AE77-9D0522C38D60

http://zoobank.org/937F3475-5EDC-4269-B1FB-623F3DC78BC9

Figures 1–5 , 58

#### Type locality.

Guyana, Region IX, Parabara, trail to mines, 2°05.095'N, 59°14.174'W, 250m.

#### Type material.

Holotype in CSBD (currently in KUNHM, see above), male labeled, “Guyana: Region IX 2°05.095'N, 59°14.174'W, 250 m Parabara, Trail to mines detrital pools in forest leg. Short, Isaacs, Salisbury 2.xi.2013; GY13-1102-01A/ SEMC1271259 KUNHM-ENT/ Holotype *Desmopachria manco* Miller, 2020 [red label with black line border].” Paratypes, 1 labeled, “Guyana: Region IX 2°52.204'N, 59°55.003'W, 124 m nr. Kusad Mts., marshy area leg. Short, Isaacs, Salisbury 27x.2013; GY13-1027-01A/ SEMC1271270 KUNHM-ENT [barcode label]/ Paratype *Desmopachria manco* Miller, 2020 [blue label with black line border].”

The paratype specimen was not dissected but it has the same color pattern, size, and other features as the holotype. It is assigned to this species even though it is from some geographic distance away.

#### Diagnosis.

**Figures 1–10.**
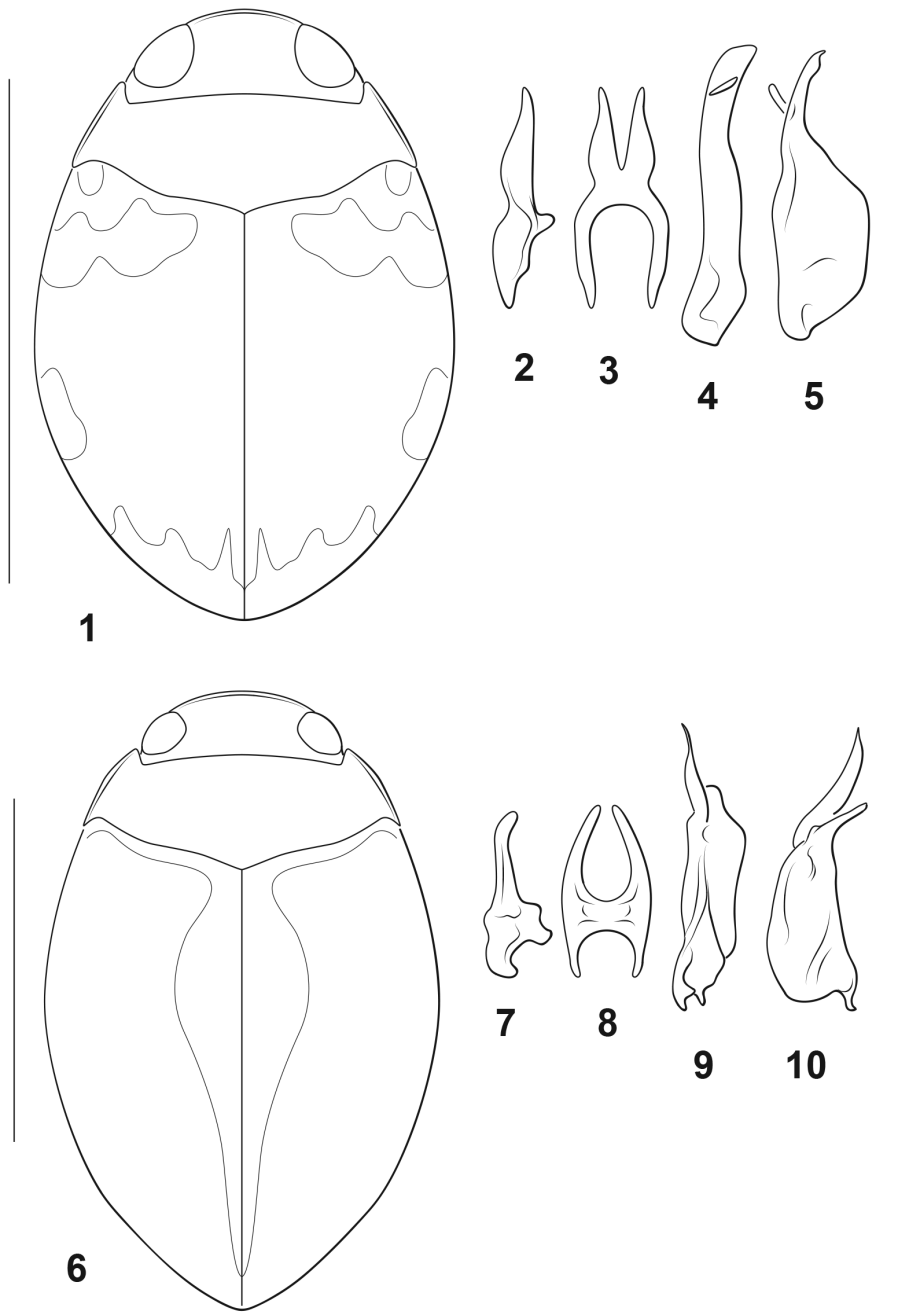
*Desmopachria* species. **1–5***D. manco***1** habitus **2–5** male genitalia **2** male median lobe, right lateral aspect **3** male median lobe, ventral aspect **4** male left lateral lobe, ventral aspect **5** male left lateral lobe, left lateral aspect **6–10***D. mortimer***6** habitus **7–10** male genitalia **7** male median lobe, right lateral aspect **8** male median lobe, ventral aspect **9** male left lateral lobe, ventral aspect **10** male left lateral lobe, left lateral aspect. Scale bars: 1.0 mm (**1, 6**).

This is an extremely small species among Dytiscidae and even among *Desmopachria* (Fig. 1; TL = 1.2–1.3 mm). The dorsal diffuse maculae on the elytra are characteristic (Fig. 1). The male genitalia include a dispositively diagnostic set of features (Figs 2–5) and place the species in the *Desmopachria convexa-signata* subgroup (see above). The median lobe is short (Figs 2, 3). In lateral aspect it is irregular in shape, medially expanded ventrally and with the apical portion slender, short, slightly curved dorsad and apically narrowly rounded (Fig. 2). In ventral aspect it is very broad, basally deeply U-shaped, apically deeply bifid, each ramus broad basally, apically narrowed and slightly curved laterad (Fig. 3). The lateral lobe in ventral aspect is elongate, broad, of subequal width throughout to a broadly truncate apex, with the subapical articulable lobe small and broad (Fig. 4). The lateral lobe in lateral aspect is very broad basally with the apex slender, subapically slightly expanded on the dorsal margin and apically sharply pointed with the subapical articulable process short and slender (Fig. 5).

#### Description.

***Measurements.***TL = 1.2–1.3 mm, GW = 0.8 mm, PW = 0.7 mm, HW = 0.4–0.5 mm, EW = 0.2 mm, TL/GW = 1.9, HW/EW = 2.0. Body round, subspherical, lateral margins continuous between pronotum and elytron (Fig. 1), dorsoventrally broad.

***Coloration*** (Fig. 1). Head and pronotum yellow. Elytron orange, with distinct but weakly margined maculae at humeral angle, anterolaterally extending to near suture, lateromedially and apically; surface not iridescent. Ventral surfaces and appendages yellow.

***Sculpture and structure.*** Head (Fig. 1) broad, anteriorly produced in rounded margin; anterior margin of clypeus margined with conspicuous, continuous, flattened bead; surface of head shiny, very finely and sparsely punctate; eyes large (HW/EW = 1.2–1.4); antennae short, scape and pedicel relatively large and rounded, flagellomere III long and slender, apically expanded, antennomeres IV–X short and broad, lobe at anterodorsal angle, antennomere XI elongate, apically pointed. Pronotum short, lateral margins short, broadly curved with narrow, even bead; surface shiny, nearly impunctate medially, more but sparsely punctate along anterior and posterior margins, punctation variable, fine to course. Elytron moderately broad, laterally broadly curved; surface impunctate. Prosternum extremely short, longitudinally compressed, medially slightly carinate; prosternal process slender anteriorly, with distinctive, small medial tubercle, apically short and broad, concave, apically broadly pointed. Metaventrite broad and evenly smoothly convex medially, surface shiny, impunctate, anteromedially with curved transverse carina between posterior margins of mesocoxal cavities; metaventrite wings extremely slender. Metacoxa with medial portion short, about 1/3 length of metaventrite medially, metacoxal lines distinctly divergent anteriorly; lateral portion of metacoxa extremely large, anteriorly strongly expanded; surface shiny, impunctate, but slightly rugulose medially. Metatrochanter large, longer than ventral margin of metafemur anterior to metatrochanter apex; legs otherwise not noticeably modified. Abdomen with surfaces shiny and smooth, impunctate.

***Male genitalia.*** Male median lobe in lateral aspect short, medially somewhat expanded on ventral margin, apically convergent to sharply angulate apex (Fig. 1). Median lobe in ventral aspect broad, base in broad “U” shape, apically deeply bifid, each ramus elongate, broad basally, and apically pointed (Fig. 2). Lateral lobe in ventral aspect moderately broad, apically gently curved laterad, with apex subtruncate, subapical articulable process oblique and flattened (Fig. 3). Lateral lobe in lateral aspect very broad basally, constricted medially, apically slender, slightly and broadly curved dorsad, apex pointed, subapically articulable lobe slender, directed ventrad (Fig. 4).

#### Etymology.

This species is named *manco*, after Manco, the younger bounty hunter in the Sergio Leone film "For a Few Dollars More".

#### Distribution.

This species is known from two localities in Guyana, Region IX (Fig. 58).

#### Habitat.

The type and paratype were collected in “detrital pools” and a “marshy area.”

### Desmopachria mortimer sp. nov.

Taxon classificationAnimaliaColeopteraDytiscidae

7E6813ED-DB86-5FA6-8860-961EAF78AB06

http://zoobank.org/F8656CD2-3D92-4B2B-94DA-5B04F8F029EC

Figures 6–10 , 59

#### Type locality.

Costa Rica, Cartago Province, Tapanti National Park, pasture by Rio Orosi, ca. 1200 m.

#### Type material.

Holotype in KUNHM, male labeled, “Costa Rica: Cartago Province Tapanti National Park: 24.v.2006 pasture by Rio Orosi: c. 1200 m leg. A.E.Z. Short, AS-06-043/ SEMC0895195 KUNHM-ENT/ Holotype *Desmopachria mortimer* Miller, 2020 [red label with black line border].” Paratypes, 41, labeled same as holotype except with different specimen barcode labels (Table 1) and each with “Paratype *Desmopachria mortimer* Miller, 2020 [blue label with black line border].”

**Table 1.** SEMC museum numbers for *D. mortimer* paratypes.

SEMC0895144	SEMC0895156	SEMC0895167	SEMC0895182
SEMC0895145	SEMC0895157	SEMC0895168	SEMC0895184
SEMC0895146	SEMC0895158	SEMC0895169	SEMC0895185
SEMC0895147	SEMC0895159	SEMC0895170	SEMC0895190
SEMC0895148	SEMC0895160	SEMC0895171	SEMC0895191
SEMC0895149	SEMC0895161	SEMC0895172	SEMC0895192
SEMC0895150	SEMC0895162	SEMC0895173	SEMC0895193
SEMC0895151	SEMC0895163	SEMC0895175	SEMC0895194
SEMC0895152	SEMC0895165	SEMC0895177	SEMC0895196
SEMC0895153	SEMC0895166	SEMC0895181	SEMC0895197
SEMC0895154			

#### Diagnosis.

This is a moderately sized, somewhat elongate species of *Desmopachria* (Fig. 1; TL = 1.7–1.9 mm). The dorsal coloration is characteristic with diffuse darker regions medially and extending laterally onto the surface of the elytron (Fig. 6). The shape of the male genitalia is diagnostic (Figs 7–10) and place the species in the *Desmopachria convexa-convexa* subgroup (see above). The median lobe is short (Figs 7, 8), and in lateral aspect it is irregularly broad basally with the apical portion slender, short and curved dorsally to a narrowly rounded apex (Fig. 7). In ventral aspect it is very broad, basally broadly U-shaped, apically deeply bifid, with each ramus slender and strongly curved medially (Fig. 8). The lateral lobe in ventral aspect is irregularly shaped, broad, with the apex narrowly truncate and with a subapical articulable lobe that extends well beyond the apex of the lateral lobe and is apically acuminate and sharply pointed (Fig. 9). The lateral lobe in lateral aspect is very broad basally with the apex slender, elongate, directed dorsad, and with the subapical articulable lobe elongate, curved and apically sharply pointed (Fig. 10).

#### Description.

***Measurements.***TL = 1.7–1.9 mm, GW = 1.1–1.2 mm, PW = 0.9–1.0 mm, HW = 0.5–0.6 mm, EW = 0.3–0.4 mm, TL/GW = 1.5–1.6, HW/EW = 1.2–1.4. Body broad but slightly elongate posteriorly, lateral margins continuous between pronotum and elytron (Fig. 6), dorsoventrally broad.

***Coloration*** (Fig. 6). Head orange, diffusely darker orange posteriorly. Pronotum orange, with darker orange region along posterior margin. Elytron orange, with diffuse, weakly margined darker regions along anteromedial margin, along elytral suture and expanded somewhat onto disc medially; surface not iridescent. Mesoventrite, metacoxa and abdominal ventrites dark orange, other ventral surfaces and appendages lighter orange.

***Sculpture and structure.*** Head (Fig. 6) broad, anteriorly produced in broadly rounded margin; anterior margin of clypeus margined with conspicuous, continuous narrow bead; surface of head shiny, very finely and sparsely punctate, punctation slightly denser posteriorly; eyes large (HW/EW = 2.0); antennae short, scape and pedicel relatively large and rounded, flagellomere III long and slender, apically expanded, antennomeres IV–X short and broad, lobe at anterodorsal angle, antennomere XI elongate, apically pointed. Pronotum short, lateral margins short, slightly curved with narrow bead, bead wider medially, slightly angulate medially; surface shiny, nearly impunctate medially, more but sparsely punctate along anterior and posterior margins, punctation variable, fine to course. Elytron moderately broad, laterally broadly curved; surface shiny, more coarsely and evenly punctate than pronotum, punctation shallow and indistinct, of variable sizes. Prosternum extremely short, longitudinally compressed, medially slightly carinate; prosternal process slender anteriorly, with distinctive, small medial tubercle, apically short and broad, concave, apically broadly pointed. Metaventrite broad and evenly smoothly convex medially, surface shiny, with very few indistinct punctures, anteromedially with curved transverse carina between posterior margins of mesocoxal cavities; metaventrite wings extremely slender. Metacoxa with medial portion short, less than 1/2 length of metaventrite medially, metacoxal lines distinctly divergent anteriorly; lateral portion of metacoxa extremely large, anteriorly strongly expanded; surface shiny, evenly but shallowly and indistinctly punctate, punctures evenly distributed. Metatrochanter large, longer than ventral margin of metafemur anterior to metatrochanter apex; legs otherwise not noticeably modified. Abdomen with surfaces shiny and smooth, nearly impunctate.

***Male genitalia.*** Male median lobe in lateral aspect irregularly shaped, apical portion slender, short, slightly curved dorsad, apically narrowly rounded (Fig. 7). Median lobe in ventral aspect very broad, basally U-shaped, apically deeply and broadly bifid, each ramus slender, apically narrowly rounded, gently curved, and convergent medially at apices (Fig. 8). Lateral lobe in ventral aspect irregularly shaped, broad, with subapical articulable lobe slender, undulate, apically sharply pointed and elongate, extending well beyond apex of lateral lobe (Fig. 9). Lateral lobe in lateral aspect broad basally, apex slender, oblique, directed dorsad, subapical articulable process slender, elongate curved and sharply pointed (Fig. 10).

#### Etymology.

This species is named *mortimer*, after Colonel Douglas Mortimer, the older bounty hunter in the film "For a Few Dollars More".

#### Distribution.

This species is known only from one locality in Costa Rica, Prov. Cartago (Fig. 58).

#### Habitat.

The only known habitat information is the type series collected in a “pasture.”

### Checklist of *Desmopachria* species in the *D. convexa* group

***Desmopachria convexa* - *convexa* species group**

**Figures 11–36.**
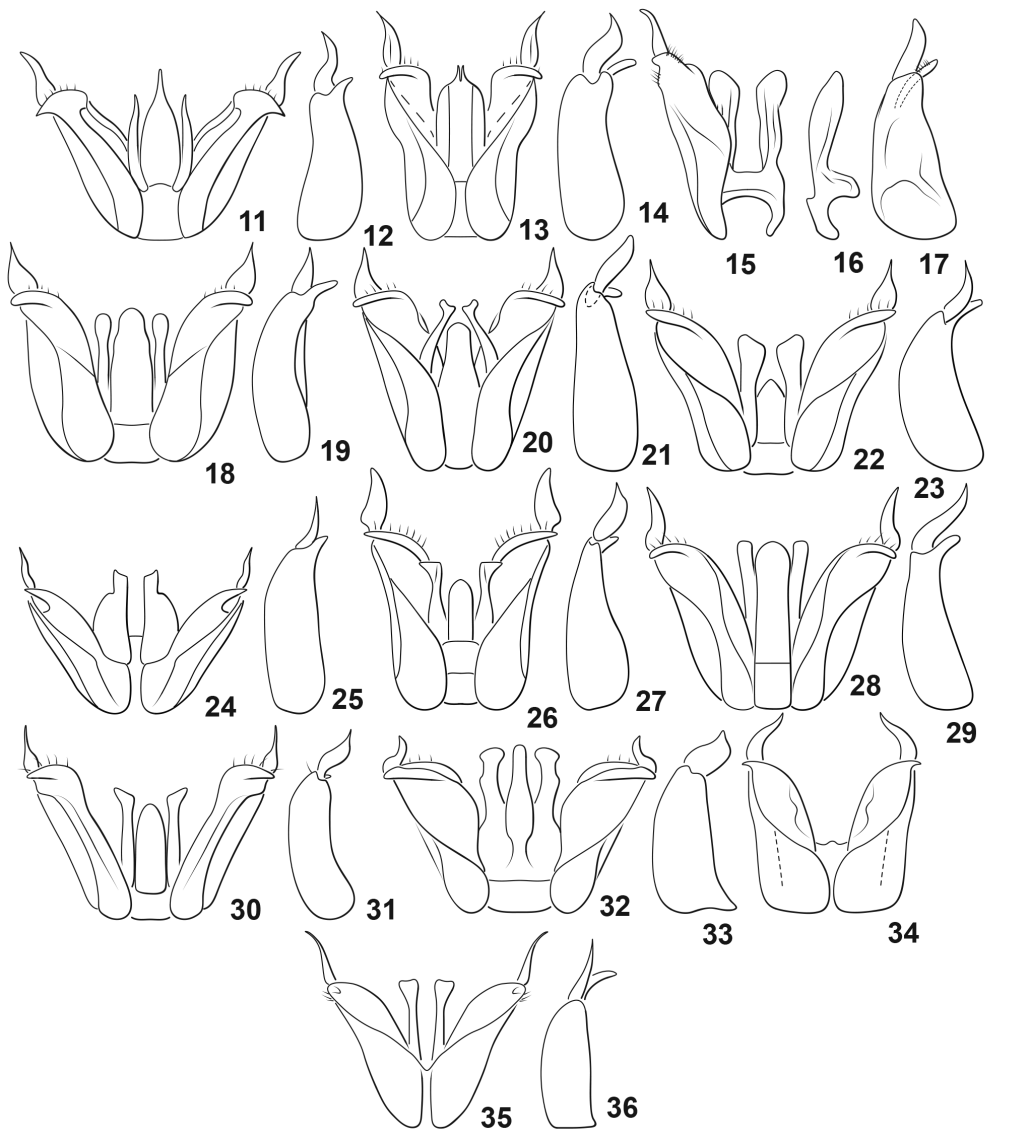
*Desmopachria* species, male genitalia. **11, 12***D. aspera***11** dorsal aspect **12** right lateral lobe, right lateral aspect **13, 14***D. cenchramis***13** dorsal aspect **14** right lateral lobe, right lateral aspect **15–17***D. challeti***15** median lobe and right lateral lobe, dorsal aspect **16** median lobe, right lateral aspect **17** right lateral lobe, right lateral aspect **18, 19***D. ciruclaris***18** dorsal aspect **19** right lateral lobe, right lateral aspect **20, 21***D. aspera***20** dorsal aspect **21** right lateral lobe, right lateral aspect **22, 23***D. defloccata***22** dorsal aspect **23** right lateral lobe, right lateral aspect **24, 25***D. glabella***24** dorsal aspect **25** right lateral lobe, right lateral aspect **26, 27***D. grana***26** dorsal aspect **27** right lateral lobe, right lateral aspect **28, 29***D. isthmia***28** dorsal aspect **29** right lateral lobe, right lateral aspect **30, 31***D. laesslei***30** dorsal aspect **31** right lateral lobe, right lateral aspect **32, 33***D. lewisi***32** dorsal aspect **33** right lateral lobe, right lateral aspect **34***D. majuscula*, dorsal aspect **35, 36***D. tarda***35** dorsal aspect **36** right lateral lobe, right lateral aspect. Redrawn from Young (1981).

*D. aspera* Young, 1981 (Florida, USA) (Figs 11, 12)

*D. cenchramis* Young, 1981 (Florida, USA) (Figs 13, 14)

*D. challeti* Miller, 2001 (Colombia) (Figs 15–17)

*D. circularis* Sharp, 1882 (Guatemala) (Figs 18, 19)

*D. convexa* (Aubé, 1838) (USA) (Figs 20, 21)

*D. defloccata* Young, 1981 (Mexico) (Figs 22, 23)

*D. glabella* Young, 1981 (Cuba) (Figs 24, 25)

*D. grana* (LeConte, 1855) (USA) (Figs 26, 27)

*D. isthmia* Young, 1981 (Panama) (Figs 28, 29)

*D. laesslei* Young, 1981 (Jamaica) (Figs 30, 31)

*D. lewisi* Young, 1981 (Jamaica) (Figs 32, 33)

*D. majuscula* Young, 1990 (Guatemala) (Fig. 34)

*D. mortimer* sp. nov. (Costa Rica) (Figs 6–10)

*D. tarda* Spangler, 1973 (Cuba) (Figs 35, 36)

***Desmopachria convexa-signata* species group**

**Figures 37–57.**
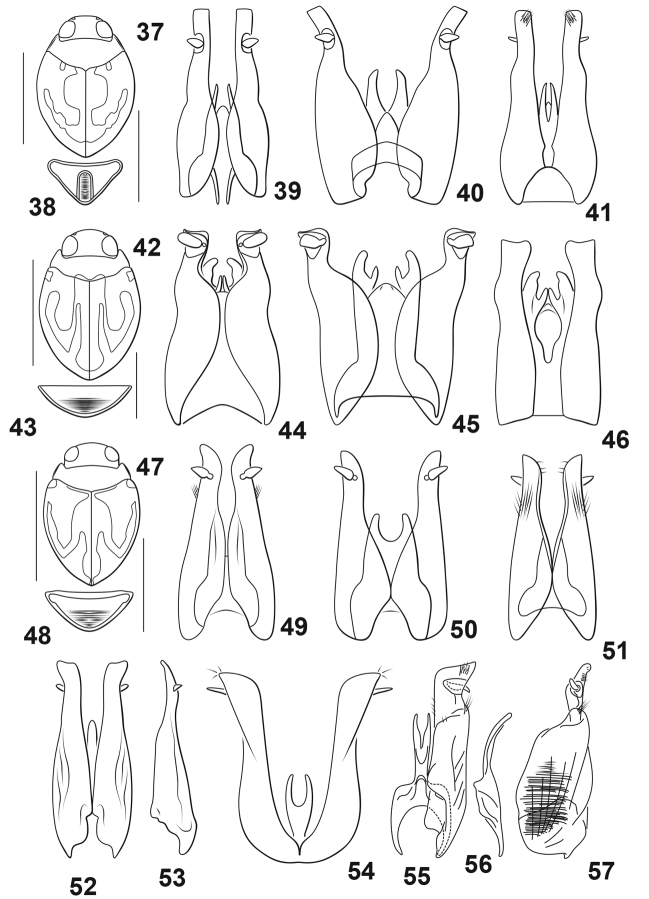
*Desmopachria* species. **37–41***D. cavia***37** habitus **38** last abdominal ventrite **39–41** male genitalia **39** male genitalia, dorsal aspect **40** male genitalia, ventral aspect **41** male genitalia, dorsal aspect with coverslip **42–46***D. manus***42** habitus **43** last abdominal ventrite **44–46** male genitalia **44** male genitalia, dorsal aspect **45** male genitalia, ventral aspect **46** male genitalia, dorsal aspect with coverslip **47–51***D. varzeana***47** habitus **48** last abdominal ventrite **49–51** male genitalia **49** male genitalia, dorsal aspect **50** male genitalia, ventral aspect **51** male genitalia, dorsal aspect with coverslip **52, 53***D. pilosa*, male genitalia **52** dorsal aspect **53** right lateral aspect **54***D. signata*, male genitalia, dorsal aspect **55–57***D. signatoides*, male genitalia **55** median lobe and left lateral lobe, dorsal aspect **56** median lobe, right lateral aspect **57** right lateral lobe, right lateral aspect. **37–51** Redrawn from Braga and Ferreira-Jr. (2010)**52, 53** redrawn from Miller (2005)**54** redrawn from Young (1990)**55–57** redrawn from Miller (2001). Scale bars: 1.0 mm (**37, 42, 47**); 0.5 mm for (**38, 43, 48**).

*D. cavia* Braga & Ferreira Jr., 2010 (Brazil) (Figs 37–41)

*D. manco* sp. nov. (Guyana) (Figs 1–5)

*D. manus* Braga & Ferreira Jr., 2010 (Brazil) (Figs 42–46)

*D. pilosa* Miller, 2005 (Peru) (Figs 52, 53)

*D. signata* Zimmermann, 1921 (Brazil) (Fig. 54)

*D. signatoides* Miller, 2001 (Bolivia) (Figs 55–57)

*D. varzeana* Braga & Ferreira Jr., 2010 (Brazil) (Figs 48–51)

**Figures 58, 59.**
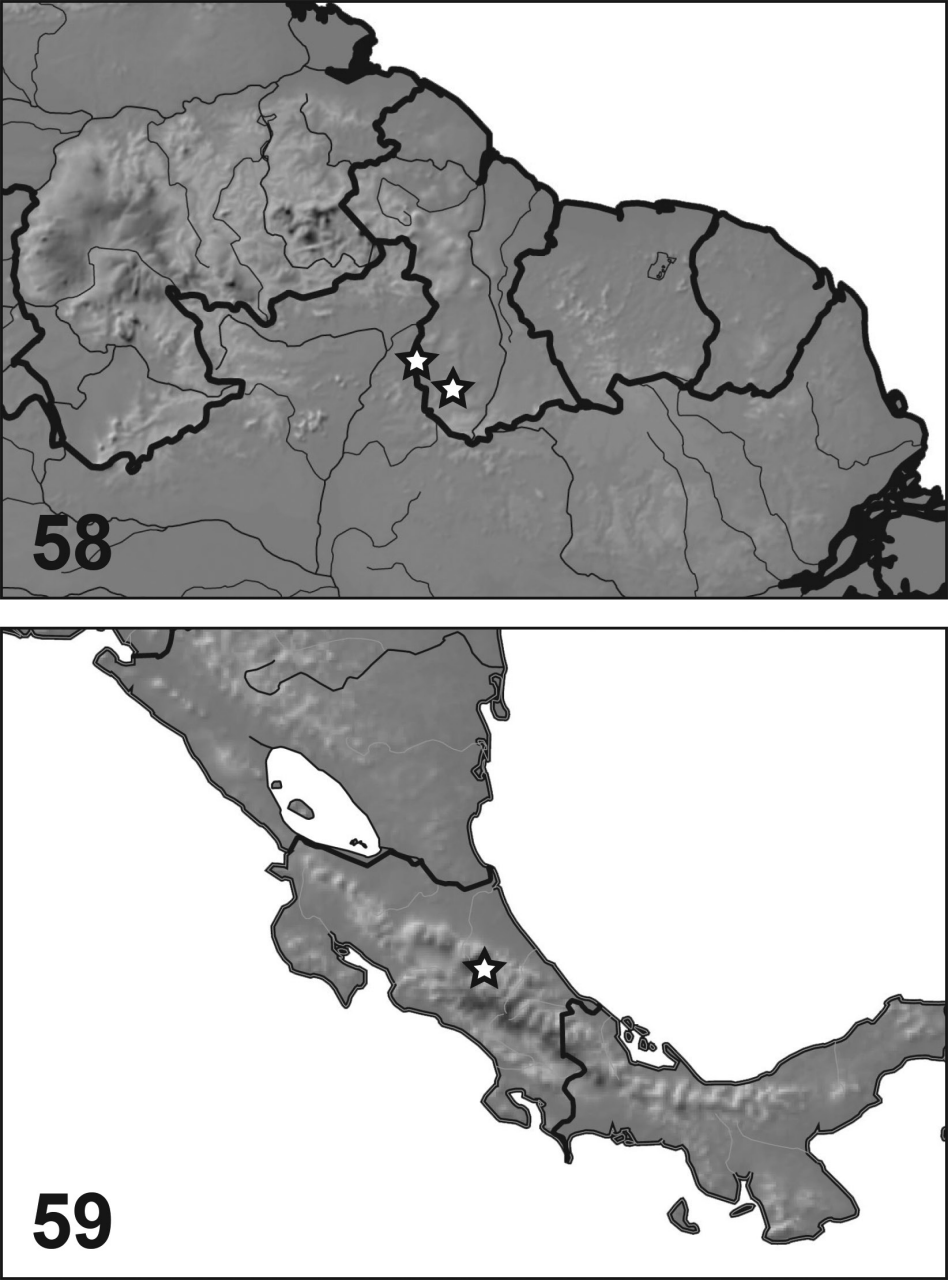
*Desmopachria* new species, distribution. **58***D. manco* (South America). **59***D. mortimer* (Central America).

## Supplementary Material

XML Treatment for Desmopachria convexa

XML Treatment for Desmopachria manco

XML Treatment for Desmopachria mortimer
